# An Adaptive Multi-Target Radar Waveform Design Based on PWS Algorithm

**DOI:** 10.3390/e22010031

**Published:** 2019-12-25

**Authors:** Bin Wang, Shumin Li, Xishi Wang, Xin Li

**Affiliations:** School of Computer and Communication Engineering, Northeastern University at Qinhuangdao, Qinhuangdao 066004, China; 1871640@stu.neu.edu.cn (S.L.); 1971780@stu.neu.edu.cn (X.W.); 1971773@stu.neu.edu.cn (X.L.)

**Keywords:** cognitive radar, information theory, probability-weighted summation (PWS), Jensen inequality, detection performance

## Abstract

Due to the uncertainty of radar target prior information in actual scenes, waveform design based on radar target prior information cannot meet the requirements of detection performance and parameter estimation. Aiming at the problem of waveform design for detecting multi-target in the presence of clutter, a linear probability-weighted summation (PWS) algorithm based on multi-target impulse response is proposed and includes the radar waveform design based on mutual information (MI) and signal-to-interference ratio (SINR) criteria. In view of the traditional water-filling algorithm, the problem of multi-target is further investigated in a new way to improve the overall performance of the system. The method makes a lot of deductions by using Jensen’s inequality, to determine the algorithm objective function and energy constraint. The simulation results show that the proposed algorithm has better detection performance and more accurate target information.

## 1. Introduction

For the problem of target recognition in cognitive radar systems, in 2015, Jiu Bo [1] proposed a method to transform the water-filling method into a universal water-filling method for clutter background, by maximizing target echo and expansion. In 2011, Fan Meimei [2] proposed that when multiple targets are angularly separable, i.e., when the targets are in different radar beams, the multi-objective waveform design problem is relatively simple, and the radar can search, detect and identify the target through multiple scans. In 2014, Wang Lulu [3,4] solved the waveform optimization method for single extended target recognition. Each of the received echoes is processed to obtain a priori information of the next transmitted waveform of each target, and is used to guide the next transmitted waveform of each target. In 2017, Goodman [5,6,7,8] proposed the task of identifying the cognitive radar waveform design target in the presence of noise, and designed the detection probability as the benchmark of performance and performance, considering the random target and the target.

The adaptive waveform design technology based on cognitive radar target recognition is a new way to realize radar target recognition technology [6]. An important constraint to the improvement of traditional radar target recognition performance is that the fixed transmitter waveform of the existing radar system is mismatched with the current state of the target and the environment. If it can adaptively, intelligently and dynamically optimize and reconfigure the transmit waveform based on the knowledge of the current target which has not been identified, the dynamic optimization and reconfiguration of the transmit waveform will greatly improve the radar system recognition performance and effectively reduce the complexity of traditional recognition algorithms [9]. The design concept of the cognitive radar system is very consistent with the above considerations. Therefore, the emergence of the cognitive radar concept and the research and development of the cognitive radar system provide a solid platform for the in-depth development of new radar target recognition technology.

The uncertainty of target characteristics is reduced by maximizing mutual information between the target echo and extended target. Then, this idea is applied to the target recognition waveform optimization problem in [10,11]. In 2010, Ref. [12] studied multiple-input multiple-output (MIMO) radar using a generalized likelihood-ratio test (GLRT) detector under the condition of synchronous phase mismatch, and to obtain the objective function based on mutual information through a large number of derivations. In 2018, Li Wang, Wei Zhu, Yunlei Zhang et al. [13] uses semi-define relaxation (SDR) technology and semi-define programming (SDP) technology to solve non-convex design problems. Simulations show that the detector has better detection performance than the GLRT detector. However, the proposed detector calculation is more complicated and burdensome, especially when the number of targets in the radar scene is large. 

However, there is no literature on the optimization and adaptive techniques of the transmitted waveforms when multiple distance-resolvable targets appear in the same beam. To solve this problem, this paper studies the optimal waveform design and adaptive technology for multiple extended target recognition in the same beam. This paper focuses on the adaptive waveform design technology for target recognition, comprehensively considers the environment, systematically analyzes and unifies the existing waveform optimization techniques for target recognition and focuses on the optimal waveform design method based on the random signal model. The content of multi-target adaptive waveform design techniques is designed to advance research and ultimately achieve true target recognition. The second section mainly discusses the theoretical basis of the research problem. The third section explains the three cases of the algorithm and the specific derivation process for each case. The fourth section realizes the feasibility of the proposed algorithm through the simulation. The final section is the conclusion, comprehensively summarizing the advantages and disadvantages of the algorithm, laying a good theoretical foundation for the later research.

## 2. Problem Formulation

### 2.1. Stochastic Multi-Targets Signal Model

Assuming there are i targets here, i=1,2,3,…,H. ∗ is convolution operation, using the traditional water-filling waveform to calculate, you can know that the target model is

(1) $$y(t)=w(t)*c(t)+w(t)*h_{i}(t)+n(t)$$

where w(t) is the transmitted waveform, c(t) is the clutter, $h_{i}(t)$ is the target impulse response, obeying a Gaussian random distribution, where $T_{y}$ denotes the duration of the echo y(t) and n(t) is Gaussian white noise. Assume that the noise power spectral density is $P_{n}(f)$; the clutter power spectral density is $P_{c}(f)$. In this model, $g_{i}(t)$ is a complex wide-sense stationary process with some power spectral density (PSD) and a(t) is a rectangular window of duration $T_{h}$. The product $g_{i}(t)=a(t)h_{i}(t)$ is a finite-duration random process with support only in $\left[0,T_{h}\right]$ and is locally stationary within $\left[0,T_{h}\right]$ since $g_{i}(t)$ is wide-sense stationary. The Fourier transform can be used to know that the time domain to frequency domain transformation is

(2) $$Y=WC+WG_{i}+N.$$

Assume that the probability of occurrence of each target is random, and the sum of the probability of occurrence of all targets is 1. The energy spectrum density of the target impulse response for each individual target is $\sigma_{g_{i}}^{2}(f)$.

### 2.2. Multi-Objective General Water-Filling Algorithm

The research of multi-target optimal waveform design based on information theory is to give the objective function from the perspective of multi-user communication, and theoretically provide a solution to the optimal waveform design problem of multi-target recognition. When the observed signal enters the receiver, the echoes of the multiple targets can be separated by the target correlation processing for each distance, and the echo of each target is processed separately. After the echo signal enters the receiver, the receiver filters of the multiple target signals have the same structure, so the noise n(t) of each target channel is considered to have the same statistical characteristics.

For the i−th target, the optimal waveform can be obtained by maximizing the MI and SINR of the received waveform $y_{i}(t)$ and the target echo $h_{i}(t)$. The expressions based on MI and SINR are Equations (3) and (4):

(3) $$\begin{array}{l} I(y_{i}(t);h_{i}(t)|w(t))=T_{y}\int_{B}\ln\left[1+\frac{{\left|w(f)\right|}^{2}\sigma_{h_{i}}^{2}(f)}{T_{y}\left\{P_{n}(f)+{\left|w(f)\right|}^{2}P_{c}(f)\right\}}\right]df \end{array}$$

(4) $$\begin{array}{l} SINR=T_{y}\int_{B}\frac{{\left|w(f)\right|}^{2}\sigma_{h_{i}}^{2}(f)}{T_{y}\left\{P_{n}(f)+{\left|w(f)\right|}^{2}P_{c}(f)\right\}}df \end{array}$$

In the multi-target case, $I(y_{i}(t);h_{i}(t)|w(t))$ (the mutual information of the received waveform and the echo) can be maximized by the multi-objective impulse response $\sigma_{h_{i}}^{2}(f)$. Where assuming that $g_{i}(t)=a(t)h_{i}(t)$ in the finite-duration time, a(t) is a window function in the corresponding time range. Random multi-objective classification and recognition algorithm based on the hypothesis testing principle was proposed by Goodman in 2017 [5]. We can know the relationship between the target recognition probability and the target energy spectrum variance is $S_{R}(f)=\sum_{i=1}^{H}P_{r}(H_{i})\sigma_{g_{i}}^{2}(f)-{\left|\sum_{i=1}^{H}P_{r}(H_{i})\sqrt{\sigma_{g_{i}}^{2}(f)}\right|}^{2}$, where $\sigma_{g_{i}}^{2}(f)$ is the energy spectrum density of the i−th target, and where $\mu_{R}(f)={\left|\sum_{i=1}^{H}P_{r}(H_{i})\sqrt{\sigma_{g_{i}}^{2}(f)}\right|}^{2}$. Therefore, the new target energy spectrum variance is $S_{R}(f)=\sum_{i=1}^{H}P_{r}(H_{i})\sigma_{g_{i}}^{2}(f)-\mu_{R}(f)$.

Before we derive the probability-weighted summation (PWS), we define $S_{R}(f)=\sum_{i=1}^{H}P_{r}(H_{i})\sigma_{g_{i}}^{2}(f)-\mu_{R}(f)$ with the given H-number of target impulse responses. Therefore, the multi-target function is derived for the MI-based and SINR-based design. Section 3 contains an explanation in detail. Finally, based on this formula, the mutual information energy spectrum variance (MIESV) algorithm is proposed. Each target is independent in the process of solving and assumes $\sum_{i=1}^{H}P_{r}(H_{i})=1$, a PWS-MI algorithm is proposed by using Jensen’s inequality [4].

Equation (3) considers each target as a random target and we propose an optimal waveform design method based on MIESV. Therefore, from the perspective of target processing, a PWS-MI optimal waveform design method is proposed. The linear probability-weighted sum of the impulse responses of multiple targets is regarded as an overall impulse response. The transmit waveform objective function of the PWS-MI algorithm is obtained based on the MI criterion.

In the multi-target cognitive radar waveform design process, the corresponding target weight is assigned corresponding probability weight according to the random allocation principle and all weighted targets are regarded as an overall target model. Based on mutual information and signal-to-interference ratio criteria, the radar optimal transmit waveform is designed under energy constraints. It is verified by detecting the probability that the proposed linear probability weighting algorithm is better than the general water-filling algorithm in detection performance under the two criteria.

## 3. Multi-Target Recognition Algorithm Based on PWS (Probability-Weighted Summation)

### 3.1. Transmit Waveform Design Based on Mutual Information

#### 3.1.1. Random Target in Noise

In this subsection, we propose solving the multi-target optimal waveform in the case of noise only. Assuming the transmit signal is essentially limited to the bandwidth B, MI can be written as

(5) $$\begin{array}{c} MIESV_{R}=T_{y}\int_{B}\ln\left[1+\frac{{\left|w(f)\right|}^{2}S_{R}(f)}{T_{y}\left\{P_{n}(f)\right\}}\right]df\\ =T_{y}\int_{B}\ln\left[1+\frac{{\left|w(f)\right|}^{2}\left\{\sum_{i=1}^{H}P_{r}(H_{i})\sigma_{g_{i}}^{2}(f)-\mu_{R}(f)\right\}}{T_{y}\left\{P_{n}(f)\right\}}\right]df\\ =T_{y}\int_{B}\ln\left[1+\frac{{\left|w(f)\right|}^{2}\left\{\sum_{i=1}^{H}P_{r}(H_{i})\sigma_{g_{i}}^{2}(f)-\sum_{i=1}^{H}P_{r}(H_{i})\mu_{R}(f)\right\}}{T_{y}\left\{P_{n}(f)\right\}}\right]df\\ =T_{y}\int_{B}\ln\left[1+\frac{{\left|w(f)\right|}^{2}\left\{\sum_{i=1}^{H}P_{r}(H_{i}){\hat{\sigma }}_{g_{i}}^{2}(f)\right\}}{T_{y}\left\{P_{n}(f)\right\}}\right]df \end{array}$$

As $\sum_{i=1}^{H}P_{r}(H_{i})=1$, applying Jensen’s inequality, as shown in Appendix A. The final PWS algorithm MI-based is

(6) $$\begin{array}{c} PWS-MI=T_{y}\int_{B}\ln\left[\sum_{i=1}^{H}P_{r}(H_{i})\cdot 1+\sum_{i=1}^{H}P_{r}(H_{i})\left\{\frac{{\left|w(f)\right|}^{2}\left\{\sigma_{g_{i}}^{2}(f)-\mu_{R}(f)\right\}}{T_{y}\left\{P_{n}(f)\right\}}\right\}\right]df\\ =T_{y}\int_{B}\ln\left[\sum_{i=1}^{H}P_{r}(H_{i})\cdot 1+\sum_{i=1}^{H}P_{r}(H_{i})\left\{\frac{{\left|w(f)\right|}^{2}\left\{\sum_{i=1}^{H}P_{r}(H_{i}){\hat{\sigma }}_{g_{i}}^{2}(f)\right\}}{T_{y}\left\{P_{n}(f)\right\}}\right\}\right]df\\ =T_{y}\int_{B}\ln\left[\sum_{i=1}^{H}P_{r}(H_{i})\left(1+\left\{\frac{{\left|w(f)\right|}^{2}\left\{\sum_{i=1}^{H}P_{r}(H_{i}){\hat{\sigma }}_{g_{i}}^{2}(f)\right\}}{T_{y}\left\{P_{n}(f)\right\}}\right\}\right)\right]df\\ \geq \sum_{i=1}^{H}P_{r}(H_{i})\left\{T_{y}\int_{B}\ln\left[1+\frac{{\left|w(f)\right|}^{2}\left\{{\hat{\sigma }}_{g_{i}}^{2}(f)\right\}}{T_{y}\left\{P_{n}(f)\right\}}\right]df\right\} \end{array}$$

where ${\hat{\sigma }}_{g_{i}}^{2}(f)=\sigma_{g_{i}}^{2}(f)-\mu_{R}(f)$, the target model is based on the PWS-MI algorithm under the energy constraint condition given by

(7) $$\begin{array}{l} \max\;\sum_{i=1}^{H}P_{r}(H_{i})\{T_{y}\int_{B}\ln(1+\frac{{\left|w(f)\right|}^{2}\left\{{\hat{\sigma }}_{g_{i}}^{2}(f)\right\}}{T_{y}\left\{P_{n}(f)\right\}})df\}.\\ s.t.\int_{B}{\left|w(f)\right|}^{2}\leq E_{w} \end{array}$$

Using this method, the paper proposes that the multi-target considered in the process are random in three cases. The algorithm before the Jensen inequality without transformation is defined as $MIESV_{R}$, and the algorithm after transformation is defined as PWS-MI.

From the above analysis, the objective function based on the $MIESV_{R}$ algorithm under the energy constraint is shown as

(8) $$\begin{array}{l} \max T_{y}\int_{B}\left[\ln[1+\frac{{\left|w(f)\right|}^{2}\sum_{i=1}^{H}P_{r}(H_{i}){\hat{\sigma }}_{g_{i}}^{2}(f)}{T_{y}\left\{P_{n}(f)\right\}}\right]]df.\\ \;s.t.\;\int_{-\infty }^{+\infty }{\left|w(f)\right|}^{2}df\leq E_{w} \end{array}$$

According to the proof $MIESV_{R}$ algorithm in Equation (8), the objective function is a concave function and the specific concave function proof is shown in Appendix B. Therefore, using the Lagrange multiplier method results in

(9) $$L({\left|w(f)\right|}^{2},\lambda )=\left\{T_{y}\int_{B}\ln\left[1+\frac{{\left|w(f)\right|}^{2}\left\{\sum_{i=1}^{H}P_{r}(H_{i}){\hat{\sigma }}_{g_{i}}^{2}(f)\right\}}{T_{y}\left\{P_{n}(f)\right\}}\right]df\right\}-\lambda \left[\int_{B}{\left|w(f)\right|}^{2}df-E_{w}\right].$$

We set $\partial^{2}L({\left|w(f)\right|}^{2},\lambda )/\partial {\left[{\left|w(f)\right|}^{2}\right]}^{2}=0$ to obtain the maximum information value of the multi-target function following as

(10) $$\frac{T_{y}\cdot \sum_{i=1}^{H}P_{r}(H_{i}){\hat{\sigma }}_{g_{i}}^{2}(f)}{T_{y}P_{n}(f)+{\left|w(f)\right|}^{2}\sum_{i=1}^{H}P_{r}(H_{i}){\hat{\sigma }}_{g_{i}}^{2}(f)}=\lambda .$$

The optimal waveform solution that maximizes the MI under the energy constraint in Equation (8) is given by Equation (11)

(11) $${\left|w(f)\right|}^{2}=\max(0,\frac{T_{y}}{\lambda }-\frac{p_{n}(f)T_{y}}{\sum_{i=1}^{H}P_{r}(H_{i}){\hat{\sigma }}_{g_{i}}^{2}(f)}).$$

The multi-target function of the optimization procedure is the PWS, and its structure is composed of the sum of multiple mutual information measures. Each target’s mutual information value is defined based on the i−th filtered spectral energy function as showed in Equation (7). The i−th filtered spectral energy function is the remnant spectral energy in which the common spectral energy $\mu_{R}(f)$ has been removed. Classification is a process of emphasizing the difference between the target signatures as opposed to their shared spectral information. Thus, it is reasonable to remove the common spectral information for target classification waveform design.

Applying Jensen inequality to derive the mathematical formula, we can determine the objective function and constraints of the PWS-MI algorithm proposed in this paper. As Equation (7) shows, it is a process based on the multi-objective linear probability-weighted summation. Unlike the $MIESV_{R}$ algorithm, this is a process of weighting the summation of the overall targets mutual information based on mutual information criterion. It is clear to see that the amplitude spectrum of the optimal waveform can be obtained. However, the optimal waveform to be solved is an H-element equation, and the optimal transmit waveform of the PWS algorithm cannot be directly obtained.

But it can be seen from the analysis that each independent multi-target satisfies the $MIESV_{R}$ algorithm, and the $MIESV_{R}$ algorithm transmit waveform can be solved. The optimal transmitted waveform spectrum ${\left|w(f)\right|}^{2}$ based on the maximum mutual information is solved by the $MIESV_{R}$ algorithm, and then the linear probability-weighted summation of all target maximum mutual information waveform spectrum is performed. The optimal transmit waveform of the PWS algorithm is obtained by computer simulation.

#### 3.1.2. MI-Based Waveform Design for Stochastic Target in Signal-Dependent Interference

Under the premise of a multi-target recognition algorithm based on mutual information in the case of noise only in Section 3.1.1, a multi-target recognition algorithm based on mutual information in the presence of clutter is proposed. The problem of solving a multi-target optimal waveform can be expressed by the following optimization problem model

(12) $$\begin{array}{c} MIESV_{R}=T_{y}\int_{B}\ln\left[1+\frac{{\left|w(f)\right|}^{2}S_{R}(f)}{T_{y}\left\{P_{n}(f)+{\left|w(f)\right|}^{2}P_{c}(f)\right\}}\right]df\\ =T_{y}\int_{B}\ln\left[1+\frac{{\left|w(f)\right|}^{2}\left\{\sum_{i=1}^{H}P_{r}(H_{i})\sigma_{g_{i}}^{2}(f)-\mu_{R}(f)\right\}}{T_{y}\left\{P_{n}(f)+{\left|w(f)\right|}^{2}P_{c}(f)\right\}}\right]df\\ =T_{y}\int_{B}\ln\left[1+\frac{{\left|w(f)\right|}^{2}\left\{\sum_{i=1}^{H}P_{r}(H_{i})\sigma_{g_{i}}^{2}(f)-\sum_{i=1}^{H}P_{r}(H_{i})\mu_{R}(f)\right\}}{T_{y}\left\{P_{n}(f)+{\left|w(f)\right|}^{2}P_{c}(f)\right\}}\right]df\\ =T_{y}\int_{B}\ln\left[1+\frac{{\left|w(f)\right|}^{2}\left\{\sum_{i=1}^{H}P_{r}(H_{i}){\hat{\sigma }}_{g_{i}}^{2}(f)\right\}}{T_{y}\left\{P_{n}(f)+{\left|w(f)\right|}^{2}P_{c}(f)\right\}}\right]df. \end{array}$$

As $\sum_{i=1}^{H}P_{r}(H_{i})=1$, using Jensen’s inequality, as shown in Appendix A. The final MI-based PWS algorithm becomes

(13) $$\begin{array}{c} PWS-MI=T_{y}\int_{B}\ln\left[\sum_{i=1}^{H}P_{r}(H_{i})\cdot 1+\sum_{i=1}^{H}P_{r}(H_{i})\left\{\frac{{\left|w(f)\right|}^{2}\left\{\sigma_{g_{i}}^{2}(f)-\mu_{R}(f)\right\}}{T_{y}\left\{P_{n}(f)+{\left|w(f)\right|}^{2}P_{c}(f)\right\}}\right\}\right]df\\ =T_{y}\int_{B}\ln\left[\sum_{i=1}^{H}P_{r}(H_{i})\cdot \{1+\left[\frac{{\left|w(f)\right|}^{2}\left\{\sigma_{g_{i}}^{2}(f)-\mu_{R}(f)\right\}}{T_{y}\left\{P_{n}(f)+{\left|w(f)\right|}^{2}P_{c}(f)\right\}}\right]\}\right]df\\ \geq \sum_{i=1}^{H}P_{r}(H_{i})\left\{T_{y}\int_{B}\ln\left[1+\frac{{\left|w(f)\right|}^{2}\left\{{\hat{\sigma }}_{g_{i}}^{2}(f)\right\}}{T_{y}\left\{P_{n}(f)+{\left|w(f)\right|}^{2}P_{c}(f)\right\}}\right]df\right\} \end{array}$$

where ${\hat{\sigma }}_{g_{i}}^{2}(f)=\sigma_{g_{i}}^{2}(f)-\mu_{R}(f)$ the target model based on the PWS-MI algorithm becomes as shown in Equation (14) under the energy constraint condition

(14) $$\begin{array}{l} \max\;\sum_{i=1}^{H}P_{r}(H_{i})\{T_{y}\int_{B}\ln(1+\frac{{\left|w(f)\right|}^{2}\left\{{\hat{\sigma }}_{g_{i}}^{2}(f)\right\}}{T_{y}\left\{P_{n}(f)+{\left|w(f)\right|}^{2}P_{c}(f)\right\}})df\}.\\ s.t.\int_{B}{\left|w(f)\right|}^{2}\leq E_{w} \end{array}$$

Based on the $MIESV_{R}$ algorithm, the multi-target function under energy constraints is

(15) $$\begin{array}{l} \max T_{y}\int_{B}\left[\ln[1+\frac{{\left|w(f)\right|}^{2}\sum_{i=1}^{H}P_{r}(H_{i}){\hat{\sigma }}_{g_{i}}^{2}(f)}{T_{y}\left\{P_{n}(f)+{\left|w(f)\right|}^{2}P_{c}(f)\right\}}\right]]df.\\ \;s.t.\;\int_{-\infty }^{+\infty }{\left|w(f)\right|}^{2}df\leq E_{w} \end{array}$$

Because the multi-target function is a concave function, the specific proof is shown in Appendix B, so you can use the Lagrange multiplier method to calculate, assuming

(16) $$L({\left|w(f)\right|}^{2},\lambda )=\left\{T_{y}\int_{B}\ln\left[1+\frac{{\left|w(f)\right|}^{2}\left\{\sum_{i=1}^{H}P_{r}(H_{i}){\hat{\sigma }}_{g_{i}}^{2}(f)\right\}}{T_{y}\left\{P_{n}(f)+{\left|w(f)\right|}^{2}P_{c}(f)\right\}}\right]df\right\}-\lambda \left[\int_{B}{\left|w(f)\right|}^{2}df-E_{w}\right].$$

We set $\partial^{2}L({\left|w(f)\right|}^{2},\lambda )/\partial {\left[{\left|w(f)\right|}^{2}\right]}^{2}=0$ to obtain the maximum information value of the multi-target function following as

(17) $$\frac{P_{n}(f)\cdot \sum_{i=1}^{H}P_{r}(H_{i}){\hat{\sigma }}_{g_{i}}^{2}(f)}{\left[T_{y}(P_{n}(f)+{\left|w(f)\right|}^{2}P_{c}(f))+{\left|w(f)\right|}^{2}\sum_{i=1}^{H}P_{r}(H_{i}){\hat{\sigma }}_{g_{i}}^{2}(f)][P_{n}(f)+{\left|w(f)\right|}^{2}P_{c}(f)\right]}=\frac{\lambda }{T_{y}}.$$

In the equation, only ${\left|w(f)\right|}^{2}$ is required, others are known or constant, where λ is a Lagrange multiplier. The simulation process requires a loop iteration to determine the specific expression of λ.

By solving, we can know

(18) $$A(f)=T_{y}\left[P_{c}{}^{2}(f)+\sum_{i=1}^{H}P_{r}(H_{i}){\hat{\sigma }}_{g_{i}}^{2}(f)\cdot P_{c}(f)\right]$$

(19) $$B(f)=2T_{y}\cdot P_{n}(f)\cdot P_{c}(f)+T_{y}\cdot P_{n}(f)\cdot (\sum_{i=1}^{H}P_{r}(H_{i}){\hat{\sigma }}_{g_{i}}^{2}(f))$$

(20) $$C(f)=T_{y}\cdot P_{n}{}^{2}(f)-\frac{T_{y}}{\lambda }\cdot \sum_{i=1}^{H}P_{r}(H_{i}){\hat{\sigma }}_{g_{i}}^{2}(f)\cdot P_{n}(f).$$

Finally, the expression ${\left|w(f)\right|}^{2}$ can be found according to the root formula

(21) $${\left|w(f)\right|}^{2}=\frac{-B(f)\pm \sqrt{B^{2}(f)-4A(f)\cdot C(f)}}{2A(f)}.$$

Since the energy spectrum is always positive, let us assume $\frac{T_{y}}{\lambda }=A$ that the final transmit waveform expression is

(22) $$\max{\left|w(f)\right|}^{2}=\max(-R(f)+\sqrt{R^{2}(f)+S(f)(A-D(f))},0)$$

where

(23) $$R(f)=\frac{\left(2P_{c}^{2}(f)+\sum_{i=1}^{H}P_{r}(H_{i}){\hat{\sigma }}_{g_{i}}^{2}(f))(P_{n}(f)T_{y}\right)}{P_{c}^{4}(f)+\sum_{i=1}^{H}P_{r}(H_{i}){\hat{\sigma }}_{g_{i}}^{2}(f)\cdot P_{c}^{2}(f)}$$

(24) $$S\left(f\right)=\frac{P_{n}(f)T_{y}\sum_{i=1}^{H}P_{r}(H_{i}){\hat{\sigma }}_{g_{i}}^{2}(f)}{P_{c}^{4}(f)+\sum_{i=1}^{H}P_{r}(H_{i}){\hat{\sigma }}_{g_{i}}^{2}(f)\cdot P_{c}^{2}(f)}$$

and

(25) $$D\left(f\right)=\frac{P_{n}(f)T_{y}}{\sum_{i=1}^{H}P_{r}(H_{i}){\hat{\sigma }}_{g_{i}}^{2}(f)}.$$

Through the solution of the $MIESV_{R}$ algorithm, the final PWS-MI is also solved accordingly. Finally, the waveform between the transmit waveform energy and the detection probability of the PWS-MI, $MIESV_{R}$ and the basic linear frequency modulation (LFM) signal is compared.

### 3.2. SINR-Based Waveform Design for Stochastic Target in Signal-Dependent Interference

Based on the same background and the random target distribution, the result is also the same as Section 3.1. The problem of solving the multi-target optimal waveform based on the SINR can be expressed by the following optimization problem model as

(26) $$\begin{array}{c} SINRESV_{R}=T_{y}\int_{B}\left[\frac{{\left|w(f)\right|}^{2}S_{R}(f)}{T_{y}\left\{P_{n}(f)+{\left|w(f)\right|}^{2}P_{c}(f)\right\}}\right]df\\ =T_{y}\int_{B}\left[\frac{{\left|w(f)\right|}^{2}\left\{\sum_{i=1}^{H}P_{r}(H_{i})\sigma_{g_{i}}^{2}(f)-\mu_{R}(f)\right\}}{T_{y}\left\{P_{n}(f)+{\left|w(f)\right|}^{2}P_{c}(f)\right\}}\right]df\\ =T_{y}\int_{B}\left[\frac{{\left|w(f)\right|}^{2}\left\{\sum_{i=1}^{H}P_{r}(H_{i})\sigma_{g_{i}}^{2}(f)-\sum_{i=1}^{H}P_{r}(H_{i})\mu_{R}(f)\right\}}{T_{y}\left\{P_{n}(f)+{\left|w(f)\right|}^{2}P_{c}(f)\right\}}\right]df\\ =T_{y}\int_{B}\left[\left\{\frac{{\left|w(f)\right|}^{2}\cdot \sum_{i=1}^{H}P_{r}(H_{i})\left\{\sigma_{g_{i}}^{2}(f)-\mu_{R}(f)\right\}}{T_{y}\left\{P_{n}(f)+{\left|w(f)\right|}^{2}P_{c}(f)\right\}}\right\}\right]df\\ =T_{y}\int_{B}\left[\left\{\frac{{\left|w(f)\right|}^{2}\cdot \sum_{i=1}^{H}P_{r}(H_{i})\left\{{\hat{\sigma }}_{g_{i}}^{2}(f)\right\}}{T_{y}\left\{P_{n}(f)+{\left|w(f)\right|}^{2}P_{c}(f)\right\}}\right\}\right]df. \end{array}$$

As $\sum_{i=1}^{H}P_{r}(H_{i})=1$, applying Jensen’s inequality, the specific proof is shown in Appendix A, and finally, PWS-SINR algorithm objective function becomes

(27) $$\begin{array}{c} PWS-SINR=T_{y}\int_{B}\left[\sum_{i=1}^{H}P_{r}(H_{i})\left\{\frac{{\left|w(f)\right|}^{2}\left\{\sigma_{g_{i}}^{2}(f)-\mu_{R}(f)\right\}}{T_{y}\left\{P_{n}(f)+{\left|w(f)\right|}^{2}P_{c}(f)\right\}}\right\}\right]df\\ \geq \sum_{i=1}^{H}P_{r}(H_{i})\left\{T_{y}\int_{B}\left[\frac{{\left|w(f)\right|}^{2}\left\{\sigma_{g_{i}}^{2}(f)-\mu_{R}(f)\right\}}{T_{y}\left\{P_{n}(f)+{\left|w(f)\right|}^{2}P_{c}(f)\right\}}\right]df\right\}. \end{array}$$

The same settings and analysis are in Section 3.1. Therefore, the PWS-SINR algorithm under energy constraints objective function proposed in this paper becomes

(28) $$\begin{array}{l} \max\;\sum_{i=1}^{H}P_{r}(H_{i})\left\{T_{y}\int_{B}\frac{{\left|w(f)\right|}^{2}\left\{\sigma_{g_{i}}^{2}(f)-\mu_{R}(f)\right\}}{T_{y}\left\{P_{n}(f)+{\left|w(f)\right|}^{2}P_{c}(f)\right\}}df\right\}\\ s.t.\int_{B}{\left|w(f)\right|}^{2}\leq E_{w} \end{array}$$

where ${\hat{\sigma }}_{g_{i}}^{2}(f)=\sigma_{g_{i}}^{2}(f)-\mu_{R}(f)$. Jensen’s inequality, which is shown in Appendix A. Therefore, the final target model based on the PWS-SINR algorithm under energy constraints becomes as follows

(29) $$\begin{array}{l} \max\;\sum_{i=1}^{H}P_{r}(H_{i})\{T_{y}\int_{B}\frac{{\left|w(f)\right|}^{2}\left\{{\hat{\sigma }}_{g_{i}}^{2}(f)\right\}}{T_{y}\left\{P_{n}(f)+{\left|w(f)\right|}^{2}P_{c}(f)\right\}}df\}.\\ s.t.\int_{B}{\left|w(f)\right|}^{2}\leq E_{w} \end{array}$$

The multi-target function based on the $SINRESV_{R}$ algorithm under energy constraints is

(30) $$\begin{array}{l} \max T_{y}\int_{B}\left(\frac{{\left|w(f)\right|}^{2}\sum_{i=1}^{H}P_{r}(H_{i}){\hat{\sigma }}_{g_{i}}^{2}(f)}{T_{y}\left\{P_{n}(f)+{\left|w(f)\right|}^{2}P_{c}(f)\right\}}\right)df.\\ \;s.t.\;\int_{B}{\left|w(f)\right|}^{2}df\leq E_{w} \end{array}$$

The derivation of Equation (30) is a concave function shown in Appendix B. The objective functions that can be calculated using the Lagrange multiplier technology

(31) $$L({\left|w(f)\right|}^{2},\lambda )=\left\{T_{y}\int_{B}\left[\frac{{\left|w(f)\right|}^{2}\left\{\sum_{i=1}^{H}P_{r}(H_{i}){\hat{\sigma }}_{g_{i}}^{2}(f)\right\}}{T_{y}\left\{P_{n}(f)+{\left|w(f)\right|}^{2}P_{c}(f)\right\}}\right]df\right\}-\lambda \left[\int_{B}{\left|w(f)\right|}^{2}df-E_{w}\right].$$

For the maximum signal to Interference noise ratio value of the objective function, we set $\partial^{2}L({\left|w(f)\right|}^{2},\lambda )/\partial {\left[{\left|w(f)\right|}^{2}\right]}^{2}=0$ to obtain

(32) $$\frac{\sum_{i=1}^{H}P_{r}(H_{i}){\hat{\sigma }}_{g_{i}}^{2}(f)\cdot P_{n}(f)}{{\left[(P_{n}(f)+{\left|w(f)\right|}^{2}P_{c}(f))\right]}^{2}}=\lambda .$$

Finally, the expression ${\left|w(f)\right|}^{2}$ can be found according to the root formula

(33) $${\left|w(f)\right|}^{2}=\frac{\sqrt{\frac{P_{n}(f)\cdot \sum_{i=1}^{H}P_{r}(H_{i}){\hat{\sigma }}_{g_{i}}^{2}(f)}{\lambda }}-P_{n}(f)}{P_{c}(f)}.$$

Since the energy spectrum is always positive, the final transmit waveform target spectrum is as follows

(34) $${\left|w(f)\right|}^{2}=\max[\frac{\sqrt{\frac{P_{n}(f)\cdot \sum_{i=1}^{H}P_{r}(H_{i}){\hat{\sigma }}_{g_{i}}^{2}(f)}{\lambda }}-P_{n}(f)}{P_{c}(f)},0].$$

Through the solution of the $SINRESV_{R}$ algorithm, the final PWS-SINR is also solved accordingly. The waveform between the transmit energy and the detection probability of the PWS-SINR, $SINRESV_{R}$ and the basic LFM signal is compared. Finally, the simulation compares the relationship between the transmit waveform energy and the detection probability of the three waveforms based on the signal-to-interference ratio, and verifies the advantages of the PWS algorithm.

## 4. Simulation and Results

We show simulation results that indicate the benefit of using PWS as waveform design measures for a random target model. In this subsection, we applied the MI-based and SINR-based algorithms in target class discrimination. The energy spectrum variance of the target and the power spectral density of the clutter are known, that is, the actual target realization is an unknown sample function generated by the PSD of the real target, as shown in Equations (2), (5) and (9); denote that the sampling frequency is 2, the transmit signal length and target length are both 31, the frequency of the signal is f∈[0,1], and the total energy is 1. The noise power spectral density $P_{n}(f)$ is 1 and the number of sampling points is 128. Assuming H=4, there are four targets that are subject to the distribution of the trigonometric function throughout the process. Figures 2, 5 and 8 show the distribution of the total target spectrum and clutter on *I* = [0, 1]. The clutter expression is a mixed trigonometric function, which is $P_{c}(f)=\left|0.2(\cos(0.3\pi (f+0.35)))+0.5(\sin(0.8\pi (f+0.3)))-0.15\right|+0.01$.

**Figure 1:**
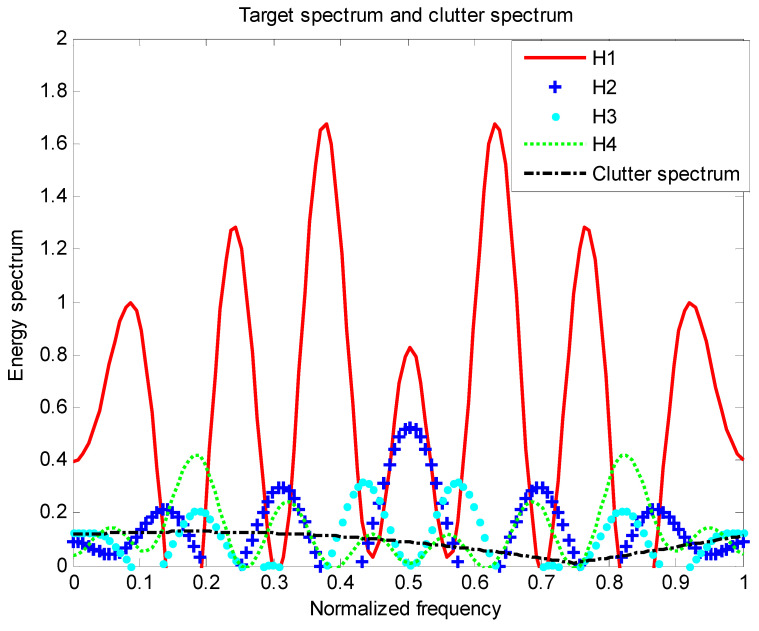
Distribution of four targets and clutter on I.

Assuming there are four targets in the research process, each of the four targets are independently distributed. The simulation process uses the total target spectrum after probability weighting. The four target spectra and the clutter spectrum are distributed as following Figure 1.

It can be seen from the basic setup simulation diagram that the four targets are independently distributed; according to the empirical setting, each target obeys the trigonometric function distribution, so that each target in the multi-target research process is not affected, which is beneficial to the target recognition.

### 4.1. Multi-Target Transmit Waveform Design Based on Mutual Information

#### 4.1.1. Random Target in Noise

In this section, we analyze the relationship between the transmitted waveform energy spectrum and the target spectrum and clutter in the case of noise only, and compare the relationship between the three waveforms detection probabilities and the energy distribution.

**Figure 2:**
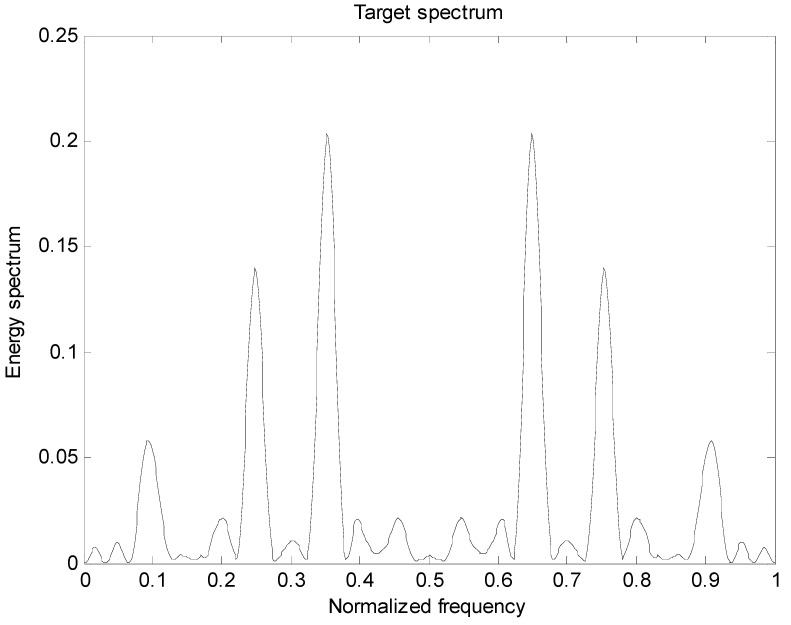
Distribution of the total target spectrum on I.

**Figure 3:**
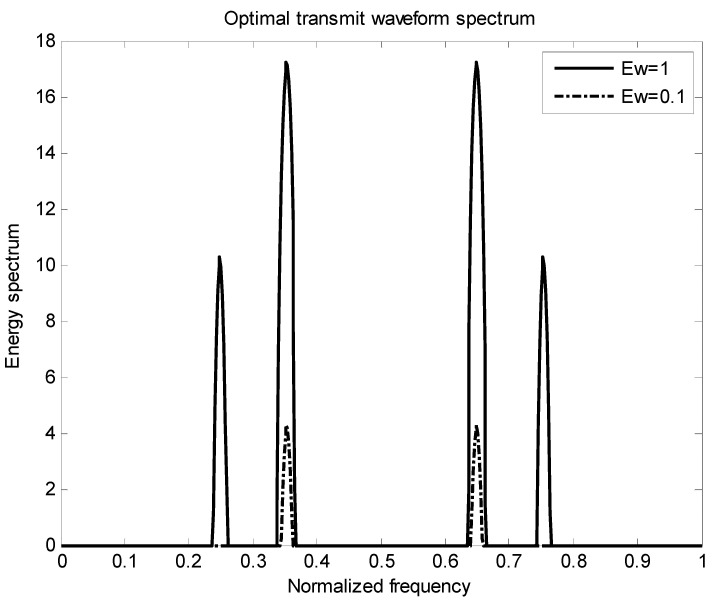
Optimal emission waveform spectrum under different energies.

Figure 2 is the energy spectral density of the optimal transmit signal in Equation (10) based on the maximum mutual information criterion in the case of noise only. It can be seen from Equation (11) that the energy spectral density of the transmit signal is determined by the targets’ impulse responses $\sum_{i=1}^{H}\sigma_{g_{i}}^{2}(f)$ and the clutter power spectral density $P_{c}(f)$. It can be seen from Figure 1 and Figure 2 that the energy spectral density (ESD) of the optimal transmit signal mainly depends on the impulse response of targets. It can be seen from Figure 3 that the optimal transmit waveform is consistent with the target impulse response waveform, and there are only some variations in the amplitude. Figure 3 shows the spectral waveform of the energy spectrum of the target transmit waveform under two energy constraints. As can be seen from the figure, under the same targets and noise conditions, the optimized waveform obtained by the optimal transmit waveform under different energy is different. It only selects the larger frequency components of the coefficients in the target spectrum, and distributes the available energy in these bands, reducing energy loss and improving energy utilization. For the case of higher energy constraint $E_{w}=1$, in order to maximize the mutual information between the target population and the received echo, the resulting optimized waveform distributes its constrained energy over a large number of frequency bands instead of only a few major frequency bands.

**Figure 4:**
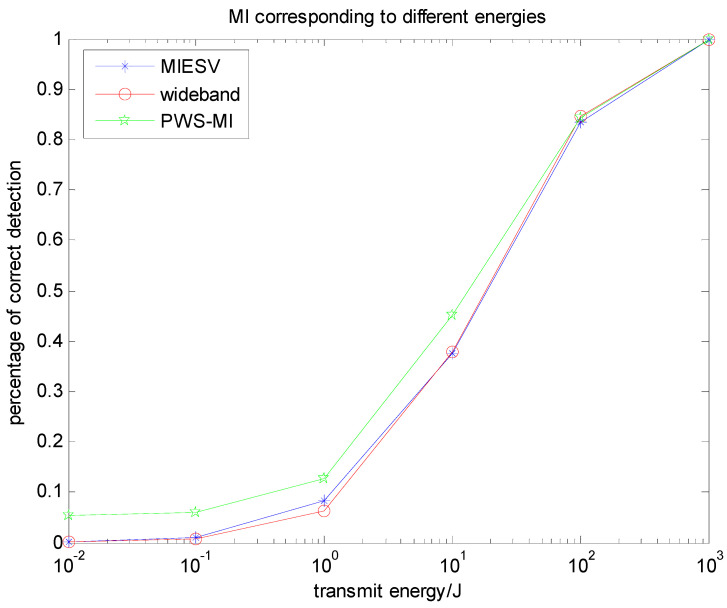
Relationship between energy spectrum and detection probability based on mutual information (MI).

Figure 4 shows a comparison of the detection probability of three kinds of waveforms based on mutual information in the case of only noise. Detection probabilities of the LFM and MIESV algorithms are very close, but the PWS-MI algorithm detection probability is still more accurate than the MIESV algorithm and better than the simplest LFM waveform, which is consistent with the previous theoretical analysis.

#### 4.1.2. MI-Based Waveform Design for Stochastic Target in Signal-Dependent Interference

In this subsection, we apply the relationship between the transmit waveform energy spectrum and the target spectrum and clutter in the presence of clutter, and compare the relationship between the three waveforms detection probabilities and the energy distribution.

**Figure 5:**
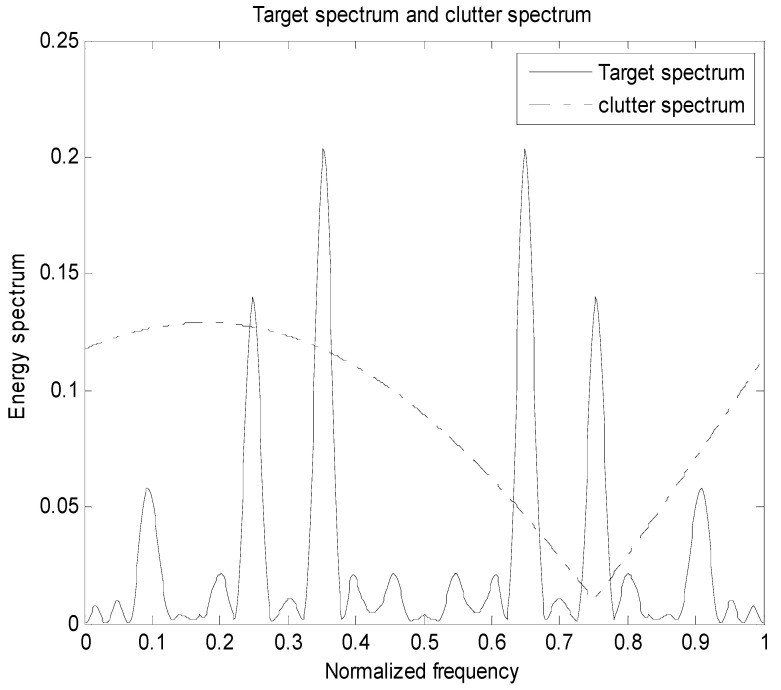
Distribution of the total target spectrum and clutter spectrum on I.

Figure 5 is a diagram showing the distribution of the total target spectrum of a plurality of targets and the clutter on [0,1] in the presence of clutter. It can be seen from Equation (17) that the energy spectral density of the transmitted signal is determined by the impulse response of targets $\sum_{i=1}^{H}\sigma_{g_{i}}^{2}(f)$ and the clutter power spectral density $P_{c}(f)$. The resulting optimized waveform concentrates energy on a few frequency bands, which ensures that the mutual information between the echo and the targets is the largest. However, due to the influence of clutter, the water-filling waveform obtained in the clutter background is different from the waveform obtained only in the noise background. When the power spectral density of the clutter is small in some frequency bands, the transmit waveform will allocate less energy in the frequency band. And in the frequency band where the clutter is stronger or the target is weaker, the energy spectrum of the transmit waveform is reduced in this band, but the trend of the target spectrum is still guaranteed. For the optimal transmit waveform spectrum, in order to compensate for the clutter, its amplitude changes compared to the target spectrum.

**Figure 6:**
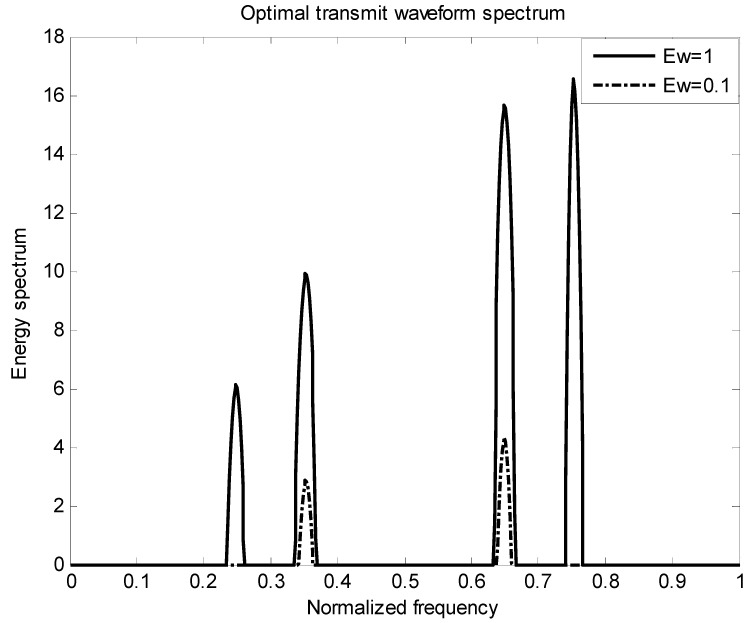
Optimal emission waveform spectrum under different energies.

Figure 6 shows the energy spectrum waveforms of two kinds of energy-constrained target transmit waveforms in the presence of clutter. It can be seen from the figure that under the same target, clutter and noise conditions, the optimal waveforms obtained under different energy transmission waveforms are different. Affected by clutter, the results obtained are different from those in Figure 3 in terms of energy allocation.

**Figure 7:**
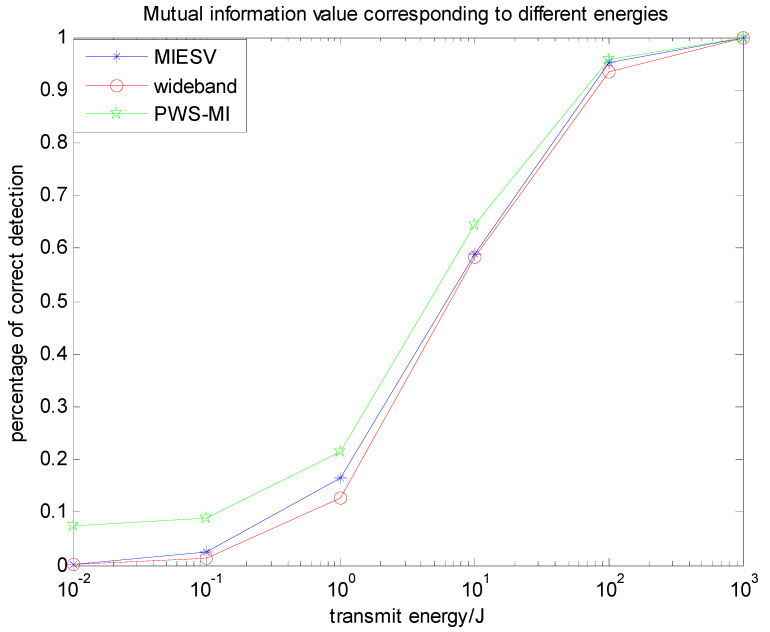
Relationship between energy spectrum and detection probability based on MI.

Figure 7 is a diagram showing the detection probability distribution of three multi-target recognition algorithms based on mutual information in the presence of clutter. The detection probabilities of the three algorithms are very close, but the PWS-MI algorithm proposed in this paper outperforms the MIESV algorithm and better than the simplest LFM waveform. The feasibility and effectiveness of the PLS algorithm proposed in this paper.

### 4.2. Multi-Target Transmit Waveform Design Based on SINR

#### SINR-Based Waveform Design for Stochastic Target in Signal-Dependent Interference

**Figure 8:**
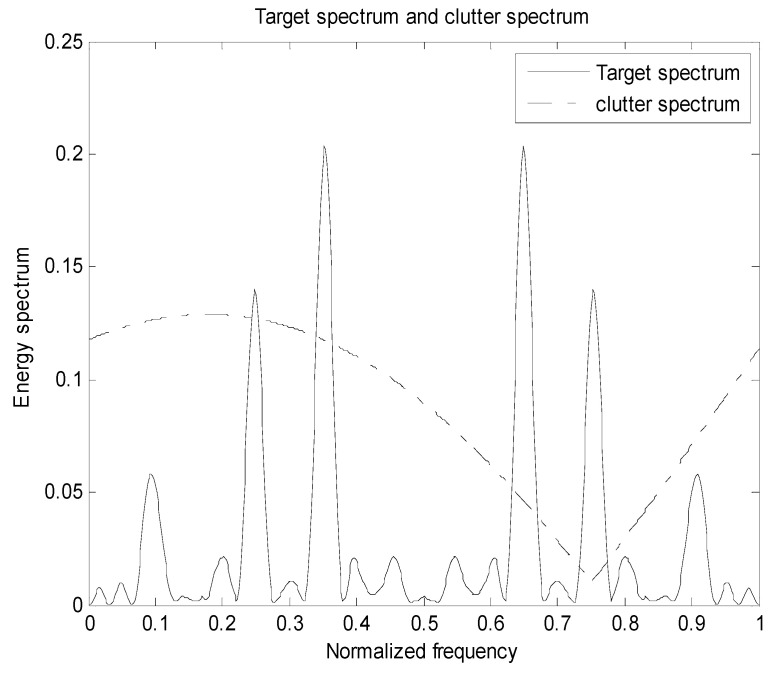
Distribution of total target spectrum and clutter spectrum on *I*.

**Figure 9:**
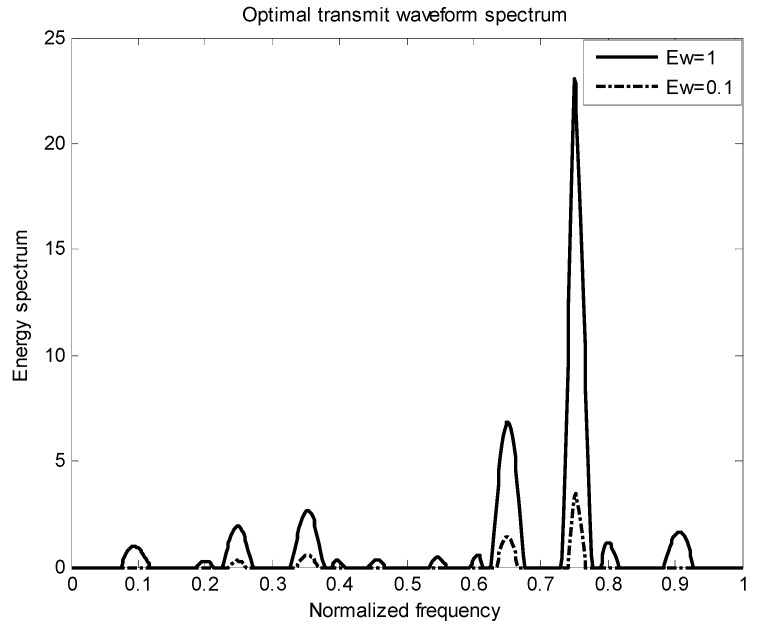
Optimal transmit waveform spectrum under different energies.

The target spectrum and the clutter spectrum based on the signal-to-interference ratio and the mutual information is the same. The clutter distribution is also a cosine-like function. The noise is still distributed in a Gaussian random white noise process. Their corresponding probabilities are randomly distributed, and the sum of the corresponding probabilities for each target is 1. Figure 8 and Figure 9 show the distribution of the total target spectrum and the clutter spectrum in *I* = [0, 1] and the distribution of the corresponding optimal transmit waveform. It can be seen from Figure 9 that the optimal transmit waveform distribution is different when the energy is different, and the larger the energy value, the more energy is allocated. Using the principle of clutter compensation, it can be seen that where there is much clutter, less energy is allocated. Experimental verification is in complete agreement with the theoretical research, and the feasibility of the proposed algorithm is verified.

**Figure 10:**
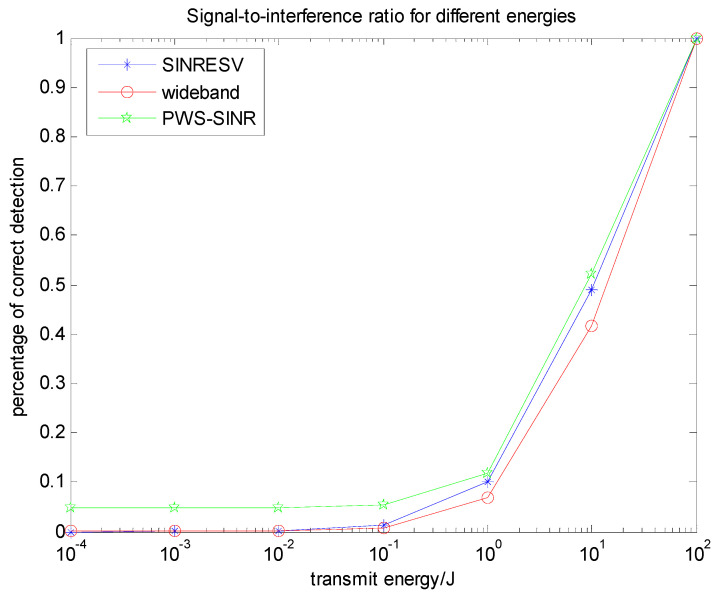
Relationship between energy spectrum and detection probability based on the signal-to-interference ratio (SINR).

Figure 10 illustrates the advantages of the PWS-SINR algorithm based on the multi-target probability weighting algorithm. For the multi-target problem, the proposed algorithm has outstanding advantages in target recognition, and the detection probability is its verification index. Under the same background and energy settings, the three waveforms SINRESV, PWS-SINR and LFM are compared. It shows that the basic trend is roughly the same, but the PWS-SINR algorithm has a better detection performance. The identification of multiple targets, reducing the uncertainty of the target, improving the overall performance of the system, has better practical value and has important research significance in target recognition.

### 4.3. Comparison of Multi-Target Based on Different Criteria

**Figure 11:**
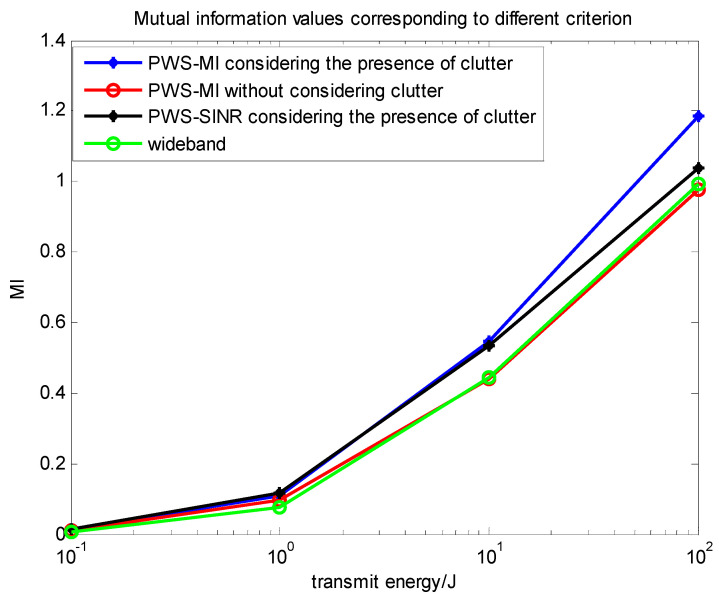
Relationship between energy spectrum and mutual information values under different criteria.

**Figure 12:**
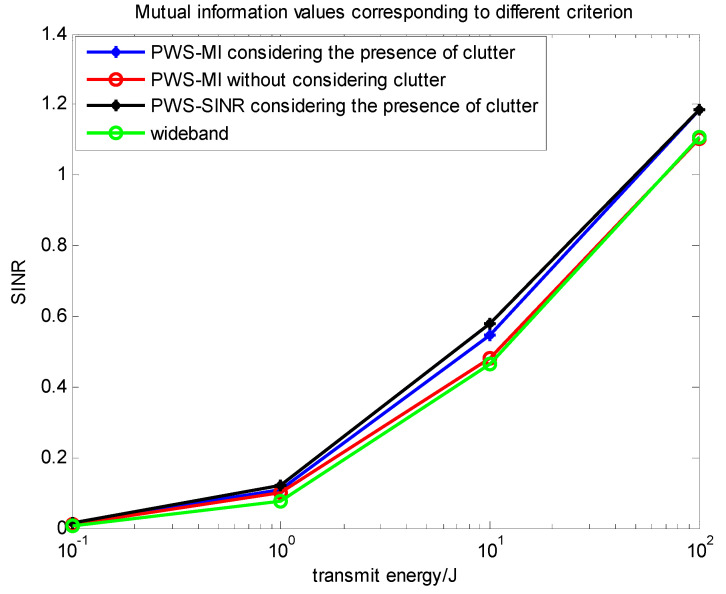
Relationship between energy spectrum and mutual information value under different criteria.

**Figure 13:**
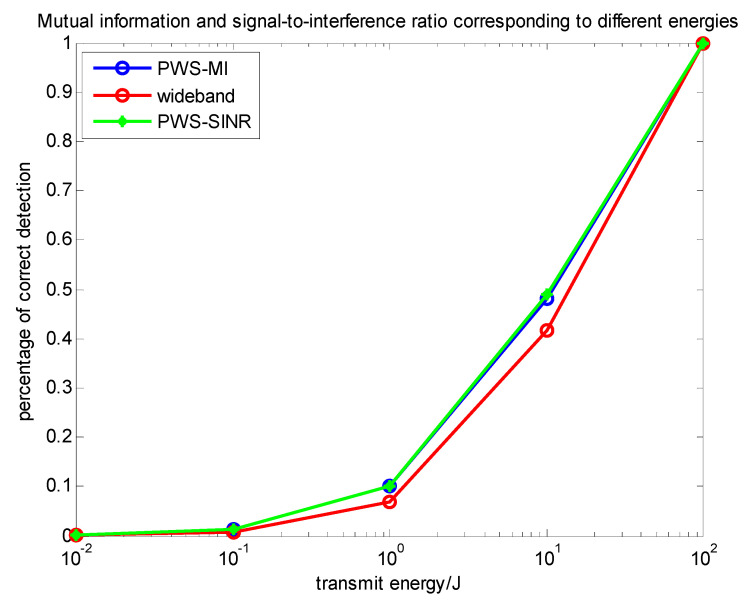
Relationship between emission waveform energy and detection probability under different conditions.

In this subsection, we comprehensively analyze the algorithms and performance involved in the above two sections. Through simulation analysis, comparative analysis in Equations (7), (14) and (29) can more clearly see the relationship between the three waveforms. Figure 11 and Figure 12 compare the above several types. The relationship between the change of the situation energy and the mutual information value and the signal-to-interference ratio, they can directly reflect the information of the target and contribute to the target recognition. Figure 13 compares the data of the PWS-MI, PWS-SINR and LFM waveforms with the change of energy in the presence of clutter. The simulation results show the pros and cons of the algorithm.

As shown in Figure 11, it verifies a comparison of the detection probabilities under the same energy constraints, the MI-maximizing waveform for signal-dependent interference outperforms the noise-only MI-maximizing waveform, the wideband impulse waveform, and the SINR-based waveform for signal-dependent interference. The wideband impulse waveform performed the poorest in extracting mutual information. It shows that in the presence of clutter, the same target and noise exist in the environment, and the constraints and energy settings are the same. In this case, we can gain higher MI and SINR in the presence of clutter. According to the theoretical derivation of the previous formulas in Equations (9) and (21), it can be concluded that the mutual information in the presence of clutter is larger than the multi-target function without considering the clutter, which is consistent with the conclusion of Goodman [5].

Figure 12 is as expected the SINR-based, clutter-compensated waveform outperforms the noise-only and the presence of clutter MI-based waveform, even the wideband impulse waveform. In fact, the presence of a clutter MI-based waveform approaches the SINR plateau of the optimum waveform in this energy-rich regime. This is due to the fact that at high energy, the system is clutter limited and while the optimum waveform places of its energy into narrow bands such that SINR is maximum, additional energy scales the target power and clutter power by approximately the same amount; hence, output SINR saturates.

It also verifies a comparison of the detection probabilities under the same energy constraints for both the presence of clutter and the case of not considering clutter. It shows that in the presence of clutter, the same target and noise exist in the environment, and the constraints and energy settings are the same. In this case, it is possible to gain a higher signal to interference ratio value in the presence of clutter. According to the theoretical derivation of the previous Equations (11) and (22), it can be concluded that the mutual information in the presence of clutter is larger than the objective function without considering the clutter, which is consistent with the conclusion of Goodman [5].

Figure 13 shows a comparison between the PWS-MI algorithm based on mutual information and the PWS-SINR algorithm based on the signal-to-interference-ratio in the context of the same target, clutter and noise settings. The detection performances of the two waveforms mentioned above along with the wideband impulse waveform are shown in the bottom panel of Figure 13. It is clear to see that the results follow the pattern of results from the target recognition experiment of the previous subsection. The clutter-compensating MI-based and SNR-based waveforms outperform the wideband impulse waveform. In this experiment, the MI-based and SNR-based waveform detection performances are very close.

## 5. Conclusions

According to the working principle of the cognitive radar closed-loop feedback system, in order to improve the performance of target recognition and detection, radar waveform based on probability-weighted summation is designed, and the single target is extended to multiple targets. A comprehensive treatment of transmit/receive waveform design matched to stochastic extended targets has been presented. We develop the multi-target waveform design in the presence of clutter and use the detection probability as a criterion to decide the performance of the target.

For a finite-duration random target, waveform designs were derived for three possible paradigms: MI-based waveform design in noise; MI-based waveform design in signal-dependent interference; SINR-based waveform design in signal-dependent interference.

In this paper, the design of radar transmitted waveform based on mutual information and signal-to-interference ratio under energy constraints and bandwidth constraints is introduced in detail. The simulation results show that the proposed PWS method performs better than the target detection probability of the LFM signal and the $ESV_{R}$ algorithm. Among them, the PWS-MI algorithm is more efficient than the algorithm that does not consider clutter after adding clutter, which verifies the effectiveness of the algorithm. Therefore, based on the PWS-MI algorithm and PWS-SINR algorithm, it is more in line with practical needs, which has certain practical significance for cognitive radar waveform design. However, how cognitive radar acquires a priori knowledge of multiple targets, noise and clutter requires further research.

Numerous examples have been performed to show the performance of optimum waveforms in various examples. We evaluate the waveforms in terms of the SINR and MI metrics used to derive the waveform as well as for a target recognition application for a limited number of target hypotheses.

## Appendix A

Jensen’s inequality

(a) If f(x) is a concave function on the interval (a,b), then for any $x_{1},x_{2},x_{3},\ldots ,x_{n}\in (a,b)$, $\lambda_{i}≻0(i=1,2,3\ldots ,n)$ and $\sum_{i=1}^{n}\lambda_{i}=1$, there is $f(\sum_{i=1}^{n}\lambda_{i}x_{i})\geq \sum_{i=1}^{n}\lambda_{i}f(x_{i})$.
(b) If f(x) is a convex function on the interval (a,b), then for any $x_{1},x_{2},x_{3},\ldots ,x_{n}\in (a,b)$, $\lambda_{i}≻0(i=1,2,3\ldots ,n)$ and $\sum_{i=1}^{n}\lambda_{i}=1$, there is $f(\sum_{i=1}^{n}\lambda_{i}x_{i})\leq \sum_{i=1}^{n}\lambda_{i}f(x_{i})$.

## Appendix B

Certificate concavity—convexity of function.

Let the function y=f(x) be continuous on *I* and have first and second derivatives in *I*, then,

(a) If there is a constant $f^{\prime \prime }(x)\geq 0$ in *I*, the pattern of f(x) on *I* is concave.
(b) If there is a constant $f^{\prime \prime }(x)\leq 0$ in *I*, the pattern of f(x) on *I* is convex.

According to the above lemma, the concavity and convexity of the objective function based on the mutual information and the signal-to-interference ratio criterion are proved.

The objective function based on mutual information (only noise) energy constraints.

(A1) $$\begin{array}{l} \max T_{y}\int_{B}\left[\ln[1+\frac{{\left|w(f)\right|}^{2}\sum_{i=1}^{H}P_{r}(H_{i}){\hat{\sigma }}_{g_{i}}^{2}(f)}{T_{y}\left\{P_{n}(f)\right\}}\right]]df.\\ \;s.t.\;\int_{-\infty }^{+\infty }{\left|w(f)\right|}^{2}df\leq E_{w} \end{array}$$

The multi-target function is continuous on *I*, with first and second derivatives in *I*.

By calculation, we can know:

(A2) $$\frac{\partial L({\left|w(f)\right|}^{2},\lambda )}{\partial {\left|w(f)\right|}^{2}}=T_{y}\cdot \ln\left[1+\frac{{\left|w(f)\right|}^{2}\sum_{i=1}^{H}P_{r}(H_{i}){\hat{\sigma }}_{g_{i}}^{2}(f)}{T_{y}\left\{P_{n}(f)\right\}}\right]-\lambda {\left|w(f)\right|}^{2}$$

(A3) $$\frac{\partial^{2}L({\left|w(f)\right|}^{2},\lambda )}{\partial {\left[{\left|w(f)\right|}^{2}\right]}^{2}}=\frac{T_{y}\cdot \sum_{i=1}^{H}P_{r}(H_{i}){\hat{\sigma }}_{g_{i}}^{2}(f)}{T_{y}P_{n}(f)+{\left|w(f)\right|}^{2}\sum_{i=1}^{H}P_{r}(H_{i}){\hat{\sigma }}_{g_{i}}^{2}(f)}-\lambda .$$

Under the energy constraint, the Lagrange multiplier λ searches within the range $0\leq \lambda \leq \max[\frac{\sum_{i=1}^{H}P_{r}(H_{i}){\hat{\sigma }}_{g_{i}}^{2}(f)}{P_{n}(f)}]$ to satisfy the constant energy value of the signal. Then it can be concluded $\frac{\partial^{2}L({\left|w(f)\right|}^{2},\lambda )}{\partial {\left[{\left|w(f)\right|}^{2}\right]}^{2}}\geq 0$.

According to the lemma, the multi-target function sought is a concave function.

The objective function based on mutual information (the presence of clutter)

(A4) $$\begin{array}{l} \max T_{y}\int_{B}\left[\ln[1+\frac{{\left|w(f)\right|}^{2}\sum_{i=1}^{H}P_{r}(H_{i}){\hat{\sigma }}_{g_{i}}^{2}(f)}{T_{y}\left\{P_{n}(f)+{\left|w(f)\right|}^{2}P_{c}(f)\right\}}\right]]df.\\ \;s.t.\;\int_{-\infty }^{+\infty }{\left|w(f)\right|}^{2}df\leq E_{w} \end{array}$$

The multi-target function is continuous on *I*, with first and second derivatives in *I*.

By calculation, we can know:

(A5) $$\frac{\partial L({\left|w(f)\right|}^{2},\lambda )}{\partial {\left|w(f)\right|}^{2}}=T_{y}\cdot \ln\left[1+\frac{{\left|w(f)\right|}^{2}\sum_{i=1}^{H}P_{r}(H_{i}){\hat{\sigma }}_{g_{i}}^{2}(f)}{T_{y}\left\{P_{n}(f){\left|w(f)\right|}^{2}P_{c}(f)\right\}}\right]-\lambda {\left|w(f)\right|}^{2}$$

(A6) $$\frac{\partial^{2}L({\left|w(f)\right|}^{2},\lambda )}{\partial {\left[{\left|w(f)\right|}^{2}\right]}^{2}}=\frac{P_{n}(f)\cdot \sum_{i=1}^{H}P_{r}(H_{i}){\hat{\sigma }}_{g_{i}}^{2}(f)}{\left[T_{y}(P_{n}(f)+{\left|w(f)\right|}^{2}P_{c}(f))+{\left|w(f)\right|}^{2}\sum_{i=1}^{H}P_{r}(H_{i}){\hat{\sigma }}_{g_{i}}^{2}(f)][P_{n}(f)+{\left|w(f)\right|}^{2}P_{c}(f)\right]}-\frac{\lambda }{T_{y}}$$

Under the energy constraint, the Lagrange multiplier λ is searched in the range $0\leq \lambda \leq \max[\frac{\sum_{i=1}^{H}P_{r}(H_{i}){\hat{\sigma }}_{g_{i}}^{2}(f)}{P_{n}(f)}]$ to satisfy the constant energy value of the signal. Then we can get $\frac{\partial^{2}L({\left|w(f)\right|}^{2},\lambda )}{\partial {\left[{\left|w(f)\right|}^{2}\right]}^{2}}\geq 0$. According to the lemma, the multi-target function sought is a concave function.

The objective function based on SINR (the presence of clutter).

(A7) $$\begin{array}{l} \max T_{y}\int_{B}\left(\frac{{\left|w(f)\right|}^{2}\sum_{i=1}^{H}P_{r}(H_{i}){\hat{\sigma }}_{g_{i}}^{2}(f)}{T_{y}\left\{P_{n}(f)+{\left|w(f)\right|}^{2}P_{c}(f)\right\}}\right)df.\\ \;s.t.\;\int_{B}{\left|w(f)\right|}^{2}df\leq E_{w} \end{array}$$

The multi-target function is continuous on *I*, with first and second derivatives in *I*.

By calculation, we can know:

(A8) $$\frac{\partial L({\left|w(f)\right|}^{2},\lambda )}{\partial {\left|w(f)\right|}^{2}}=T_{y}\cdot \frac{{\left|w(f)\right|}^{2}\sum_{i=1}^{H}P_{r}(H_{i}){\hat{\sigma }}_{g_{i}}^{2}(f)}{T_{y}\left\{P_{n}(f){\left|w(f)\right|}^{2}P_{c}(f)\right\}}-\lambda {\left|w(f)\right|}^{2}$$

(A9) $$\frac{\partial^{2}L({\left|w(f)\right|}^{2},\lambda )}{\partial {\left[{\left|w(f)\right|}^{2}\right]}^{2}}=\frac{\sum_{i=1}^{H}P_{r}(H_{i}){\hat{\sigma }}_{g_{i}}^{2}(f)\cdot P_{n}(f)}{{\left[(P_{n}(f)+{\left|w(f)\right|}^{2}P_{c}(f))\right]}^{2}}-\lambda$$

Under the energy constraint, the Lagrange multiplier λ is searched in the range $0\leq \lambda \leq \max[\frac{\sum_{i=1}^{H}P_{r}(H_{i}){\hat{\sigma }}_{g_{i}}^{2}(f)}{P_{n}(f)}]$ to satisfy the constant energy value of the signal. Then we can get $\frac{\partial^{2}L({\left|w(f)\right|}^{2},\lambda )}{\partial {\left[{\left|w(f)\right|}^{2}\right]}^{2}}\geq 0$. According to the lemma, the multi-target function sought is a concave function.
